# Should Starch Metabolism Be a Key Point of the Climacteric vs. Non-climacteric Fruit Definition?

**DOI:** 10.3389/fpls.2020.609189

**Published:** 2020-12-02

**Authors:** Christian Chervin

**Affiliations:** University of Toulouse, Toulouse INP, INRA, CNRS, ENSAT, GBF, LRSV, Castanet-Tolosan, France

**Keywords:** starch, synthesis, degradation, fleshy fruit, climacteric definition

## Introduction

The usual definition of the difference between climacteric and non-climacteric fruit relies on the fact that climacteric fruit ripens with concomitant increases of respiration and ethylene production, whereas barely any change in these two metabolisms occurs in non-climacteric fruit (Cherian et al., 2014). These authors list a series of climacteric fruit, such as tomato, banana, apple and mango, and a series of non-climacteric fruit such as strawberry, melon and grape. I think melon is a particular case, with climacteric and non-climacteric cultivars (Obando-Ulloa et al., 2009; Saladié et al., 2015), and this will not be detailed here. Other fruits have such climacteric and non-climacteric cultivars within a same species, for example Asian pears (Itai and Fujita, 2008) and plums (Minas et al., 2015). There have been many other reviews and articles over the last decade, regarding the differences between climacteric and non-climacteric fruit classes (Paul et al., 2012; Osorio et al., 2013; Saladié et al., 2015; Farcuh et al., 2017; Fuentes et al., 2019), but none pointed out that starch accumulation or breakdown could be a cornerstone in the definition of these two fruit classes.

A quick data review, as detailed below, shows that most climacteric fruit accumulate starch before the onset of ripening, then starch is broken down to soluble sugars after the inception of ripening, whereas in the non-climacteric fruit the starch content drops very rapidly after anthesis, and they accumulate mainly soluble sugars throughout development and ripening. This big difference leads to different harvest strategies: climacteric fruit can be picked early, and the starch reserve will be converted to sugars over postharvest stages, whereas the non-climacteric fruit should be picked when the desired soluble sugar level is reached.

However, starch metabolism is rarely mentioned as a key difference between climacteric and non-climacteric fruit. Osorio et al. (2012) suggested that the regulation of starch synthesis may be part of this difference, when comparing climacteric (tomato) and non-climacteric (pepper) fruit transcripts around the onset of ripening.

Thus, I will first list starch contents in some climacteric and non-climacteric fruit, then I will review rapidly the starch synthesis and the starch breakdown metabolisms in plants, and finally I will discuss research perspectives.

## Starch Level Over Fruit Development in Various Species

**Figure 1:**
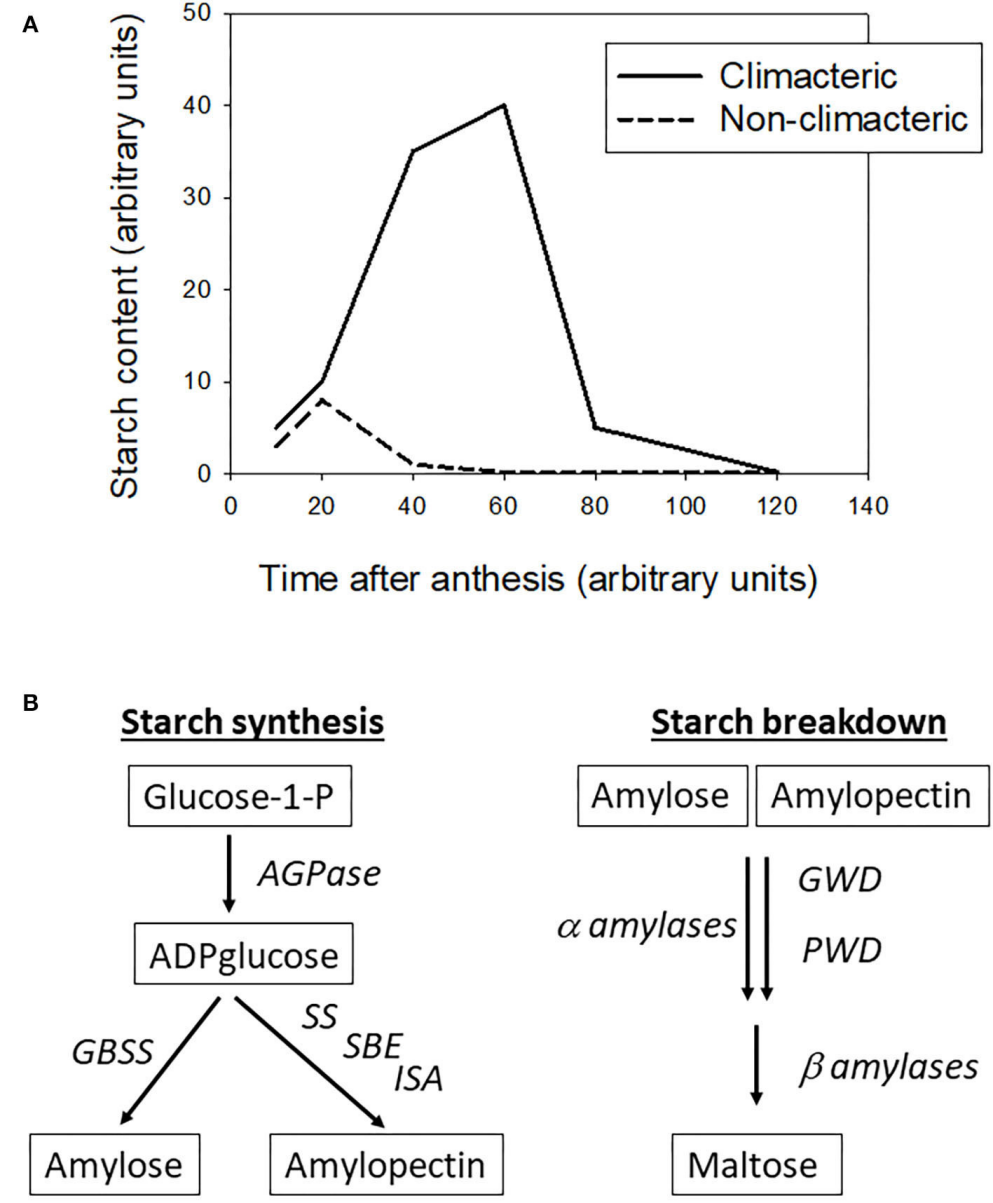
**(A)** Changes in starch accumulation over fruit development and ripening in climacteric and non-climacteric fruit (adapted from various references listed in the text). **(B)** Main steps of starch synthesis and degradation in plants, AGPase stands for ADP-glucose pyrophosphorylase, GBSS for granule-bound starch synthase, SS for starch synthase, SBE for starch branching enzyme, ISA for isoamylase, GWD for glucan water dikinase, and PWD for phosphoglucan water dikinase (adapted from various references listed in the text).

The following data are summarized in Figure 1A. In tomato, the starch content rises up to 10 mg to 20 mg/g_FW_ up to breaker stage, then drops around 0.1 mg/g_FW_ when the fruit ripens (Schaffer and Petreikov, 1997; Petreikov et al., 2009; Hou et al., 2019). In banana, the starch content is relatively high reaching 100–300 mg/g_FW_ before harvest, while the starch content in ripe fruit drops below 150 to <10 mg/g_FW_, depending the cultivar (Cordenunsi-Lysenko et al., 2019). In apple flesh the starch content can reach 20–25 mg/g_FW_ 90–120 days after anthesis, according the cultivars, then drops to levels below 0.5 mg/g_FW_ in ripe fruit (Ohmiya and Kakuishi, 1990; Brookfield et al., 1997). In mango the starch accumulates up to 60 mg/g_FW_ at harvest, then drops to <5 mg/g_FW_ in 10 days of ripening (Simão et al., 2014).

Regarding the non-climacteric fruit, the pattern of starch content is clearly different. In strawberry the starch content drops rapidly from 15 to <1 mg/g_FW_ 20 days after anthesis in developing fruit (Souleyre et al., 2004). In grape berry flesh, the starch accumulation over development and ripening is very limited, with a rapid drop from 0.5 mg/g_FW_ to stable concentrations below 0.1 mg/g_FW_, as soon as the berry reaches 20 days after anthesis up to harvest (Zhu et al., 2017). However, these authors showed that both strawberries and grapes accumulated large amount of soluble sugars, instead of starch, during the fruit development period.

## Starch Synthesis and Breakdown in Plants

Starch synthesis in plants has been reviewed (Kötting et al., 2010; Geigenberger, 2011; Pfister and Zeeman, 2016) among other works. Starch is composed of amylose and amylopectin fractions. It is a relatively simple pathway, as shown in Figure 1B, with three main steps: (1) production of ADP-glucose from glucose-1-P by an ADPglucose pyrophosphorylase (AGPase), (2) production of amylose from ADP-glucose by a granule-bound starch synthase (GBSS), or (3) production of amylopectine fromADP-glucose by a series of reactions driven by starch synthase (SS), starch branching enzyme (SBE) and isoamylase-type debranching enzyme (ISA), sometimes called debranching enzyme.

Linked to the starch synthesis, Centeno et al. (2011) showed that alterations of malate metabolism in tomatoes led to altered levels of starch accumulation, though regulation of the redox status of the AGPase.

Starch degradation is more complex than starch synthesis, as it follows different pathways according to the plant organ where they occur (Kötting et al., 2010; Zeeman et al., 2010). The glucose polymers *in vivo* are degraded mainly by β-amylases and to a lesser extent by α-amylases. The activities of these latter are regulated by the level of phosphorylation or de-phosphorylation of the glucose chains, performed by glucan water dikinase (GWD) and phosphoglucan water dikinase (PWD). For detailed mechanisms, see the review articles cited above.

## Discussion and Perspectives

From general knowledge, outlined in Figure 1A, it is clear that starch accumulation pattern is different between climacteric and non-climacteric fruit classes. This could be further reinforced by studies on a broader range of fleshy fruit, to confirm that starch accumulation pattern is an essential difference between both fruit classes. As mentioned above, Osorio et al. (2012) suggested that starch synthesis may be a key step differentiating the climacteric from the non-climacteric, thus I performed a quick literature search on this step, regarding some climacteric and non-climacteric fruit.

Robinson et al. (1988) showed that AGPase activity in developing tomatoes was closely related to starch accumulation, for which Petreikov et al. (2009) showed this enzyme is a limiting step. In banana, there is a study focused on the AGPase family, with phylogeny and expression details (Miao et al., 2017), but there is only one stage of fruit development. AGPase proteins were found in all developing stages of strawberry (Souleyre et al., 2004), but no starch accumulated. In grapes, the data about starch accumulation in fruit is scarce. There is one study about AGPase expression in inflorescences (Sawicki et al., 2015), but not in berries. What about in other fleshy fruit species?

Nowadays with increasing availability of large data sets, regarding the transcriptomes or the proteomes, further research regarding the starch synthesis pathway could be initiated comparing some model fruit. Regarding tomato, one tool has recently been published, TomExpress (Zouine et al., 2017). It regroups a wide array of RNAseq studies, and allows to search for expression patterns of all tomato genes. For banana, there is the Banana Genome Hub, containing a transcriptomics search tool (Droc et al., 2013). For apple, there is a similar web site, called AppleMDO, published recently by Da et al. (2019). For collecting grape RNAseq data and making them searchable, a new platform has been created, called Grape-RNA (Wang et al., 2020) and for Citrus sinensis a recent data basis has been created too (Feng et al., 2019). Mining such databases and others, for other fruit species, with a focus on the few starch synthesis genes, by comparing developing stages of climacteric and non-climacteric fruit, would probably generate new insight into differences between these two fruit classes, and may reinforce the fact that starch should be a cornerstone of the definition of climacteric vs. non-climacteric. This could lead to new research.

Regarding starch degradation, the mining of such large data sets may also reveal some differences between these two fruit classes, that have not yet been studied. I believe more is to be uncovered in the coming decade, regarding starch metabolism in climacteric vs. non-climacteric fruit.

## Author Contributions

CC wrote the article and created the illustration.

## Conflict of Interest

The author declares that the research was conducted in the absence of any commercial or financial relationships that could be construed as a potential conflict of interest.
